# Investigation of Solvent-Assisted In-Mold Bonding of Cyclic Olefin Copolymer (COC) Microfluidic Chips

**DOI:** 10.3390/mi13060965

**Published:** 2022-06-18

**Authors:** Qiang Li, Bingyan Jiang, Xianglin Li, Mingyong Zhou

**Affiliations:** 1 College of Mechanical and Electrical Engineering, Central South University, Changsha 410083, China; 203711016@csu.edu.cn (Q.L.); jby@csu.edu.cn (B.J.); 173711076@csu.edu.cn (X.L.); 2 State Key Laboratory of High performance Complex Manufacturing, Central South University, Changsha 410083, China

**Keywords:** microfluidic chip, cyclic olefin copolymer (COC), injection molding, in-mold bonding, solvent bonding

## Abstract

The bonding of microfluidic chips is an essential process to enclose microchannels or microchambers in a lab-on-a-chip. In order to improve the bonding quality while reducing the fabrication time, a solvent-assisted bonding strategy was proposed to seal the microchannels immediately after the cover sheet and substrate chip was injection molded in a single mold. Proper organic solvents were selected and the influences of solvent ratios on the surface roughness, microchannel morphology, and contact angle of microfluidic chips were investigated. When the solvent bonding was integrated in the mold, the influences of solvent volume fraction, solvent dosage, bonding pressure, and bonding time on the bonding quality were analyzed. Results show that the solvent cyclohexane needs to be mixed with isopropanol to reduce the dissolution effect. Solvent treatment is suggested to be performed on the cover sheet with a cyclohexane volume fraction of 70% and a dose of 1.5 mL, a bonding pressure of 2 MPa, and a bonding time of 240 s. The bonding strength reaches 913 kPa with the optimized parameters, while the microchannel deformation was controlled below 8%.

## 1. Introduction

Microfluidic chips, as a platform for implementing microfluidic technology for fluidic reactions, separations, detection, and other operations, can achieve the main functions of large, multifunctional biochemical laboratories [1]. It has a broad application prospect in the fields of biopharmaceuticals, chemical analysis, and medical testing. Compared with traditional analysis systems, less reagent consumption and detection time are required in microfluidic devices, which can meet the increasing demands for the point-of-care testing. Recently, microfluidic chips have also been used in detecting the novel coronaviruses [2,3,4]. The microfluidic chip is directly oriented to the major needs of human society, and the demand is increasing year by year.

Plenty of research have shown that microfluidic chips made with polymer materials have excellent processability. They can be manufactured with high design freedom and mass manufacturing capability [5,6]. Polymers used in microfluidics are mainly transparent thermoplastics such as polymethylmetacrylate (PMMA), cyclo-olefin-copolymer (COC), cyclo-olefinpolymer (COP), polycarbonate (PC), and polystyrene (PS). Among these thermoplastics, the COC and COP materials have outstanding optical characteristics and extremely low permeability for water vapour. Furthermore, they withstand polar organic solvents like isopropanol and acetone that are frequently used in operation on the surface of the material. Owing to these advantages, the COC/COP microfluidic chips are widely used in applications in bio-chemical and life sciences. Injection molding technology is a promising alternative to fabricate polymer microfluidic chips in scale-up production with low cost [7]. After the substrate with microchannels is injection molded, the substrate and the cover sheet need to be bonded tightly for further application. Otherwise, the bonded microfluidic chip with insufficient bonding quality often has defects such as leakage and channel blockage, which affect the analytical process in the microfluidic chip. Common bonding methods include thermal bonding [8,9,10], solvent bonding [11,12,13], laser bonding [14], adhesive bonding [15], and ultrasonic bonding [16].

Thermal bonding is widely used in the sealing process of polymer microfluidic chips due to the advantages of stability and high bonding strength. Mekaru [17] used thermal bonding technology to seal the microfluidic chips in a vacuum environment and studied the effects of bonding temperature and duration on the tensile strength. The results showed that the increase in both bonding temperature and duration increased the tensile strength of the microfluidic chips. Qu et al. [18] optimized the thermal bonding process of microfluidic chips with the goal of bonding strength and bonding percentage at the interface. However, thermal bonding of microfluidic chips is often completed on hot press equipment at a temperature close to its glass-transition temperature (*T_g_*), which could result in deformation in the microchannels and prolong the processing time [19,20]. From the author’s previous research, a novel thermal bonding strategy called in-mold bonding was proposed to shorten the manufacturing cycle [21]. In this strategy, both injection molding and thermal bonding process are sequentially integrated into a single mold. The mold temperature controller at the injection stage can provide a stable temperature during the bonding process. However, it still needs to find a balance between the bonding strength and the deformation in the microchannels that can be induced by the applied temperature and pressure.

Solvent bonding is another method that has received extensive attention in the microfluidic chip fabrication process. Polymer substrate is dissolved in organic solvent with solubility relatively close to the substrate. After that, polymer chains are mobile and can easily diffuse across the solvation layer, forming an entanglement layer and resulting in a tight bonding [22,23]. Faghih and Sharp [24] investigated the influences of solvent phases and mixing ratios on the bonding quality of PMMA microfluidic chips. The results showed that maximum bonding strength was achieved when the solvent ratio of liquid phase dichloromethane and isopropanol was 2:8. The transmittance of the microfluidic chip changed the least after the treatment with gas-phase dichloromethane. Keller et al. [25] prepared an adhesion layer on the surface of a COC chip to achieve bonding by a 3 min solvent immersion that allowed for the biocompatible bonding of protein-patterned COC, showing a bonding strength well above 744 kPa. However, for specific polymer materials, it is necessary to select a suitable solvent or mixtures to achieve the desired result in order to avoid possible damage in the microstructure. In addition, the processing parameters of the solvent bonding need to be carefully controlled. Otherwise, the organic solvent will cause channel distortion and dissolution on the surface of polymer materials [23].

The combination of thermal bonding and solvent bonding would probably be an ideal choice in the fabrication of microfluidic chips. Assisted with solvent exposure, thermal bonding can achieve very high bonding strength [19,20]. In this study, in order to shorten the manufacturing time while optimizing the bonding quality, a solvent-assisted in-mold bonding method was proposed, especially for the scale-up production of microfluidic chips. The replication-based fabrication process and back-end process of microfluidic chips can be realized in a single mold. In this work, the proper organic solvents were determined by compatibility testing based on the principle of solvent solubility. The influences of solvent ratios on the surface roughness, microchannel morphology, and contact angle of microfluidic chips were investigated. Since the solvent bonding is integrated into the mold, the effects of the solvent volume fraction, applied volume, bonding pressure, and bonding time on the bonding quality were analyzed and optimized. This study aims to achieve the high-quality, short-period integration of fabricating techniques for microfluidic chips in mass production.

## 2. Experiments and Methods

### 2.1. Injection Molding and Solvent-Assisted In-Mold Bonding

**Figure 1:**
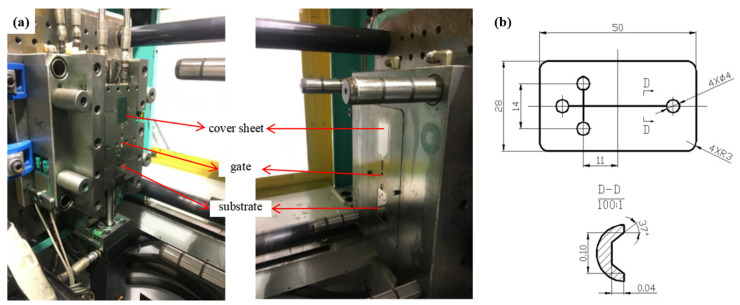
(**a**) The integrated mold with the function of both injection molding and the in-mold bonding process, (**b**) top view of the substrate of the microfluidic chip (unit mm).

**Figure 2:**
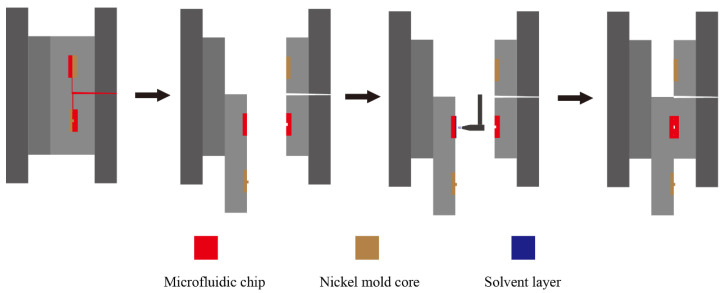
Schematic diagram of injection molding and in-mold solvent-assisted bonding.

An injection molding machine (Allrounder 370S, Arburg, Lossburg, Germany) was used to fabricate the microfluidic chip, with the integrated mold assembled on the machine, as shown in Figure 1a. It has the characteristics of synchronous molding of the substrate and cover sheet, automatic gate breaking, and sliding alignment of the movable template, which together form the injection molding and in-mold bonding system [26]. A typical electrophoresis chip with cross-channels was designed in this work. The structure of the substrate with a thickness of 0.8 mm is shown in Figure 1b. The cover sheet has the same dimension as the substrate in both width and length, while the thickness is 0.6 mm. The process of manufacturing microfluidic chips with the integrated mold is shown in Figure 2. Firstly, the substrate and the cover sheet of microfluidic chip are simultaneously molded. After the mold is opened, the dynamic template then moves down to make sure the cover sheet and substrate are at the same height coordinate. Next, the organic solvent is sprayed on the surface of the microfluidic chip by a robotic manipulator and the mold is closed again. Since the solvent has a relatively low boiling point, the majority of the solvent evaporates rapidly after contacting the surface of the hot microfluidic chip, thus forming a thin layer of solvent. The bonding process of the chip is completed by the injection molding machine with a certain holding pressure and mold temperature.

### 2.2. Solvent Selection in Solvent-Assisted Bonding

During the solvent-assisted bonding process, the organic solvent is sprayed on the surface of the microfluidic chip. The attached solvent gradually penetrates the polymer layer and dissolves the polymer. Therefore, polymer chains can diffuse across the interface more easily between the adjoining substrates due to the dissolution.

Solvent bonding is achieved mainly through the dissolution of the polymer. Therefore, the selection of a proper solvent is critical. The introduction of suitable solvents can change the surface properties of polymers, enhance the molecular chain movement at low temperatures, and achieve the bonding of chips. Nevertheless, if the solvent has a strong dissolving ability, it may cause serious damage to the chip surface and destroy the microstructure on the substrate chips. Thus, the selected solvent must be able to dissolve the polymer to improve the diffusion of molecular chains on the surface of the polymer. On the other hand, it must ensure that it does not cause damage to the microstructure. The cyclic olefin copolymer (COC, Topas 5013L-10) material is a high flow, internally lubricated injection molding grade with a heat distortion temperature of 130 °C, while the glass transition temperature (*T_g_*) is 134 °C. In the solvent-assisted bonding process, the selected solvent should match the material properties of the COC. The principle of similar solubility parameters is adopted, as shown in Table 1.

According to the Hansen solubility parameter approach, the total energy of vaporization is composed of three main interactions: nonpolar atomic interactions (dispersion forces), inherent molecular interactions (polar forces), and attractions among the molecules due to hydrogen bonds (hydrogen-bonding forces) [27,28]. As shown in Table 1, the solubility parameters of cyclohexane and COC are relatively close, with similar polarity and less toxicity. Therefore, the cyclohexane was chosen as the organic solvent for bonding. However, it is known that the cyclohexane has a strong ability to dissolve the COC surface [29,30]. In order to prevent the microchannels from being destroyed during the bonding process, the volume fraction of the cyclohexane solution needs to be appropriately reduced. From our preliminary tests, it was found that isopropanol has almost no impact on the surface of COC and is a clean solvent that can remove impurities on the polymer surface. Thus, the isopropanol was chosen to be mixed with cyclohexane as the solvent.

### 2.3. Polymer–Solvent Compatibility

To avoid the excessive solubilization of COC by pure cyclohexane, a single-factor experiment was designed to analyze the effect of different volume fractions on the surface roughness, contact angle, and surface morphology of the injection-molded chips. The solubility parameter $\delta_{m}\;$of the solvent after mixing is related to the volume fraction of the mixed solvent, which can be obtained from Equation (1) [31]:

(1) $$\delta_{m}=\varphi_{1}\delta_{1}+\varphi_{2}\delta_{2}$$

where $\varphi_{1},\varphi_{2}$ is the volume fraction of the two solvents, and $\delta_{1},\delta_{2}$ is the solubility parameter of the two solvents. Since each of the three components of a solvent mixture is a linear function of composition, the composition value to be used in calculating solubility parameters for solvent mixtures is the volume fraction (φ) for each component [31]. According to Table 1, the value of adding isopropanol to cyclohexane is to add polar and hydrogen bonding forces to the blend, since cyclohexane has essentially none. The volume fraction of cyclohexane is determined according to the solubility parameters of COC, as shown in Table 2.

#### 2.3.1. Surface Roughness

Due to the strong solubility of cyclohexane for COC, dissolution may occur on the surface of the chip after solvent treatment, causing unevenness of the surface. To observe the surface roughness, an optical surface profiler (WYKO NT9100, Veeco Metrology Inc., Plainview, NY, USA) was used to examine the solvent-treated samples.

#### 2.3.2. Microchannel Morphology

The surface of the chip will be dissolved after solvent treatment, and the substrate surface of the chip with microstructure may cause defects such as channel deformation, which affects the result of analysis and detection. The morphology of the microchannels was observed by the digital microscope (VHX-5000, Keyence, Osaka, Japan).

#### 2.3.3. Contact Angle

A contact angle measuring instrument (JC200D, Powereach, ShangHai, China) was used to inspect the surface of the sample. The change of the contact angle reflects the surface wetting performance of the solvent-treated cover chip, which helps to analyze the hydrophobicity of the channel surface.

### 2.4. Bonding Quality Characterization

Currently, the bonding quality is mainly judged by bonding strength, sealing performance, and microchannel morphology, etc. Single-factor experiments were designed to investigate the effects of parameters such as solvent volume fraction, dosage, bonding pressure, and time on the bonding quality of microfluidic chips, as shown in Table 3. In this work, the solvent dosage is the total volume of the solvent mixture that was sprayed on the surface. The baseline level for the bonding process was as follows. The volume fraction of cyclohexane was 70%; the solvent dosage was 1 mL; the processing surface was the cover sheet; the bonding pressure was 3 MPa; the bonding time was 240 s. The bonding temperature was set at a constant value of 100 °C, much lower than the *T_g_*, at which the COC chip can hardly be sealed via conventional thermal bonding.

**Figure 3:**
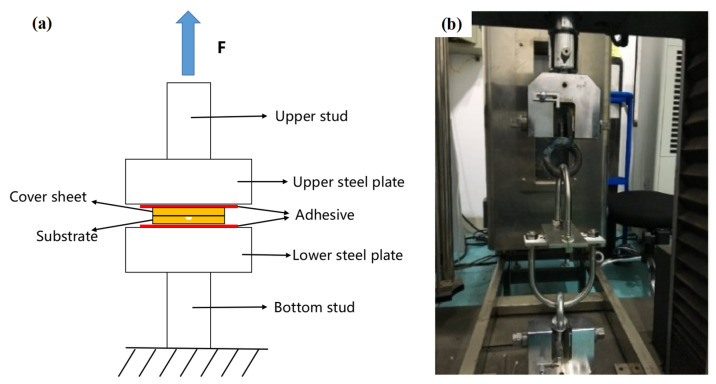
The test method of bonding strength: (**a**) the tensile strength test schematic and (**b**) the image of experimental setup. (F: force).

The tensile strength test schematic was designed according to the standard IEC62047-9. The back of the bonded microfluidic chip was polished with sandpaper, cleaned, and dried with isopropanol and deionized water. Then, the epoxy resin (Araladite 2011, Huntsman, Salt Lake City, UT, USA) was applied as an adhesive to the specific position of the metal fixture in an oven at 80 °C for 2 h. After the test sample was cooled down, it was clamped in the universal testing machine (CMT4204, MTS Systems, Eden Prairie, MN, USA) with a tensile rate of 0.5 mm/min, as shown in Figure 3. When the tension reaches a certain strength, the substrate and cover sheet are pulled apart due to insufficient bonding force, the tension transient occurs, and the machine stops working. The maximum tensile force at this point is called the fracture force (*F_c_*). The bonding strength ($\sigma_{c}$) of the chip can be calculated by Equation (2).

(2) $$\sigma_{c}=\frac{F_{c}}{A}$$

## 3. Results and Discussion

### 3.1. Analysis of Polymer–Solvent Compatibility

**Figure 4:**
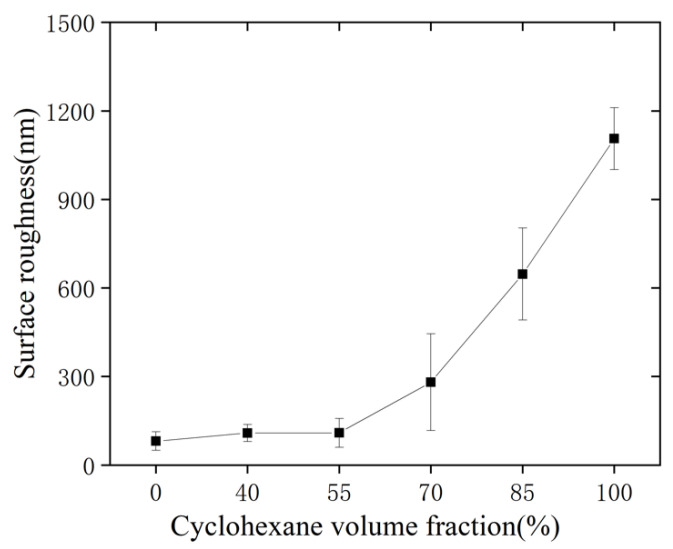
The surface roughness with different cyclohexane volume fractions.

**Figure 5:**
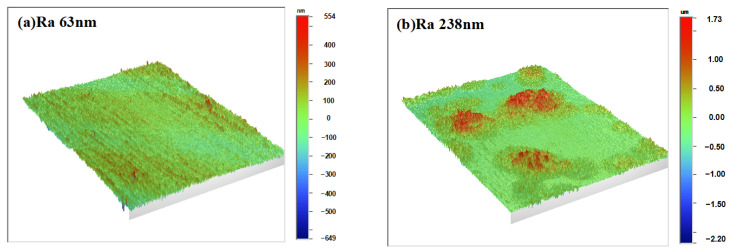
The surface roughness on the (**a**) pristine and (**b**) treated surface with the cyclohexane volume fraction of 70%.

The surface roughness of the solvent-treated microfluidic chip at different cyclohexane volume fractions is presented in Figure 4. When the volume fraction of cyclohexane is lower than 55%, the change of surface roughness is not obvious. Otherwise, the surface roughness increases rapidly. The roughness in microscope for cyclohexane volume fraction of 0% and 70% are shown in Figure 5. It is demonstrated that the cyclohexane volume fraction has a great impact on the chip surface roughness. In addition, the solubility of cyclohexane to COC is relatively weak. Moreover, the solvent evaporates quickly after spraying, which further weakens the influence on the surface quality. As the volume fraction of cyclohexane increases, the solubilization ability gradually strengthens. The surface roughness also increases since the surface is being dissolved. The solvent with pure cyclohexane shows the strongest solubilization ability. The surface roughness of the treated chips reaches a maximum value of 1106 ± 105 nm.

**Figure 6:**
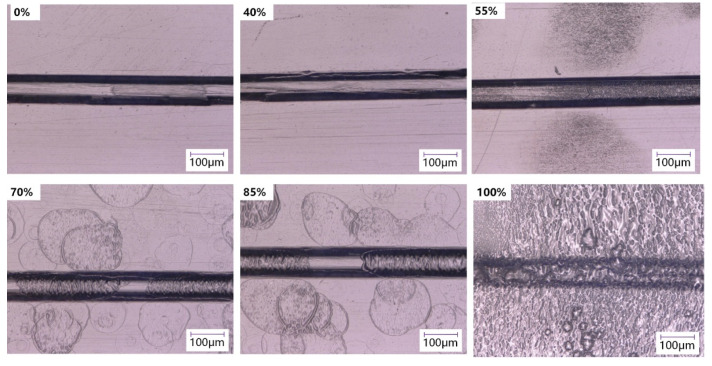
The microchannel morphology with different cyclohexane volume fractions.

After the chip substrate was treated with different volume fractions of organic solvents, the surface morphology of the microchannels was investigated, as illustrated in Figure 6. When the volume fraction of cyclohexane was below 55%, the surface was relatively smooth and no obvious damage was found in the microchannels. With the further increase in volume fraction, the surface of the microfluidic chip is dissolved to a certain extent. Unevenness and partial deformation of the microchannels can be observed while the overall structure is still in a good condition. However, the treatment with pure cyclohexane causes plenty of pits on the chip surface, which affects the integrity of the microchannels. Combined with the analysis of surface morphology and roughness, it can be found that pure cyclohexane or a high-volume fraction of cyclohexane has excessive solubility to COC and is not suitable as the solvent treatment on the substrate surface for bonding.

**Figure 7:**
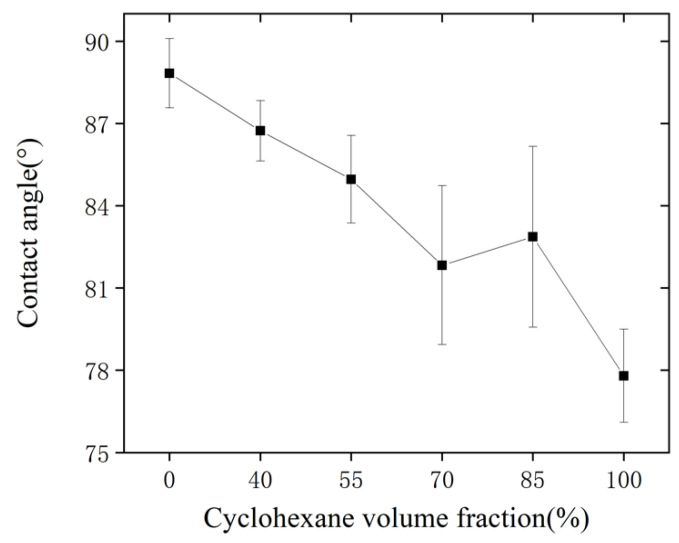
The influence of cyclohexane volume fraction on the contact angle of the treated surface.

The contact angle of solvent-treated microfluidic chips at different cyclohexane volume fractions is shown in Figure 7. With the increase in the cyclohexane volume fraction, the overall contact angle of the chip surface shows a decreasing trend. As the volume fraction of cyclohexane is higher, the surface of the COC chip becomes rougher due to being dissolved. At the same time, the molecular chains become easier to move due to the dissolution effect, therefore increasing the surface energy. However, the decrease in contact angle is still in a small range, from 88.8 ± 1.26° to 77.8 ± 1.26°. It is indicated that the breakage in chemical bonds and reorganization did not occur on the chip surface after solvent treatment; the main effect comes from the dissolution of the COC polymer.

### 3.2. Analysis of In-Mold Solvent-Assisted Bonding Quality

Firstly, the influence of the treating side on the bonding strength is analyzed. The bonding strength reaches 986 kPa when both substrate and cover sheet are treated, while the bonding strength is 864 kPa and 827 kPa on the condition that only the substrate or the cover sheet is treated, respectively. Although the bonding strength is slightly decreased, when the solvent is applied on the cover sheet, the surface of the cover sheet is dissolved. Moreover, the microstructure on the substrate is less affected and the channel remains intact. To ensure the integrity of the microchannels of the microfluidic chip, only the cover sheet is treated in the following studies.

**Figure 8:**
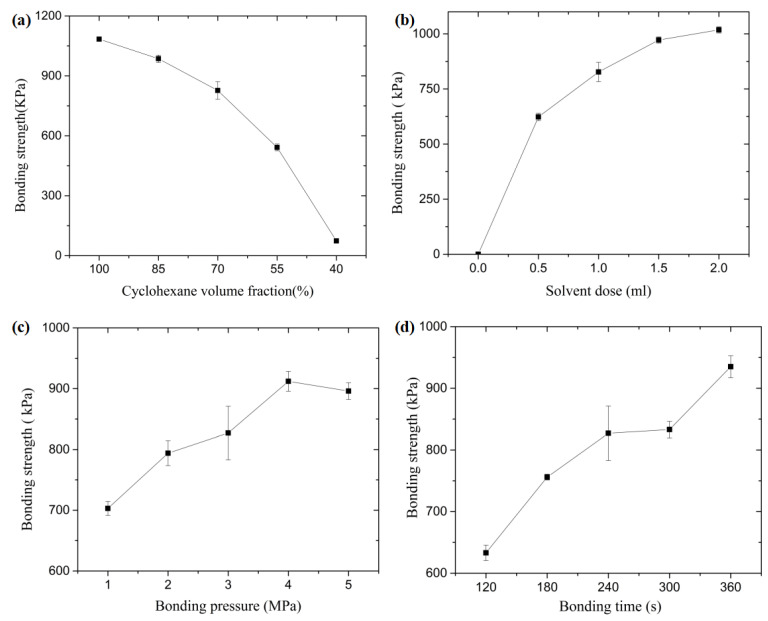
The effect of (**a**) cyclohexane volume fraction, (**b**) solvent dose, (**c**) bonding pressure, and (**d**) bonding time on the bonding strength.

The effect of the cyclohexane volume fraction on the bonding strength of the microfluidic chip is described in Figure 8a. With the reduction of cyclohexane volume fraction from 100% to 40%, the bonding strength decreases from 1084 ± 11.7 kPa to 74 ± 12 kPa. It is known that pure cyclohexane is more soluble for COC material, while isopropanol has almost no effect on the COC surface. In mixed solvents, as the volume fraction of cyclohexane decreases, the solubility of the solvent in the polymer decreases as well. There are relatively less molecular chains that can participate in diffusion movement, leading to a decrease in bonding strength. This impact is amplified to a certain extent when the volume fraction is low; the bonding strength decreases from 827 ± 44 kPa to 542 ± 16.9 kPa when the volume fraction is reduced from 70% to 55%. More obviously, when below 55%, the bonding strength is significantly reduced to 74 kPa. Due to low bonding strength, it may lead to the separation of the substrate and the cover sheet. The influence of solvent dose on the bonding strength of the microfluidic chip is presented in Figure 8b. Without solvent treatment, the chip does not bond at a bonding temperature of 100 °C. With the introduction of solvent, the bonding strength increases to 623 kPa. When the dose is increased from 0.5 mL to 2 mL, the bonding strength increases from 623 ± 17 kPa to 1018 ± 14.5 kPa. The solvent dose mainly controls the degree of dissolution of the COC material. The higher the dose of solvent, the more solute molecules it contains, and the more COC is dissolved. This means that after the solution is sprayed on the chip, the cyclohexane molecules will gradually penetrate the interior of the polymer, which will increase the adsorption and diffusion movements at the bonding interface, thus providing strong bonds. However, excessive solvent dosage may lead to the polymer being excessively dissolved. In this case, some dissolved polymer may be squeezed out of the bonding interface under the bonding pressure. In addition, there is also a risk that the polymer will be squeezed into the microchannel. Therefore, a reasonable control of the solvent treatment dose is also a guarantee to ensure successful bonding.

The effect of bonding pressure on the bonding strength of the microfluidic chip is illustrated in Figure 8c. The bonding strength increases from 703 ± 11.4 kPa to 912 ± 16.3 kPa when the bonding pressure increases from 1 MPa to 5 MPa, and then decreases to 896 ± 13.7 kPa. In thermal bonding, the main function of the bonding pressure is to enable the substrate and cover sheet to overcome the surface unevenness and the distance between them to be reduced to the extent where the polymer molecular chains can easily undergo adsorption or diffusion. The role of bonding pressure is similar in solvent-assisted in-mold bonding. Due to the existence of the solvent, even at low temperatures and low pressure, the polymer can still fill the gap at the contact interface, thus achieving a tight fit between the substrate and the cover sheet. However, when the pressure is too high, some of the dissolved polymers can spill out of the bonding interface. The surface of the chip becomes softer by the effect of the solvent, which is more likely to produce flow deformation under pressure. As the pressure increases, the flowing polymer will slowly squeeze into the microchannels, causing deformation or even blockage of the microchannels. Thus, the bonding pressure should not be too high. Figure 8d shows the influence of bonding time on the bonding strength of the microfluidic chip. The bonding strength is 633 ± 12.5 kPa at a bonding time of 120 s. As the time increases to 360 s, the bonding strength increases to 935 ± 11.7 kPa. Like the thermal bonding process, the source of bonding strength in solvent-assisted in-mold bonding relies mainly on intermolecular diffusion, adsorption, and entanglement. The molecular chains at the polymer surface are more active than usual after solvent treatment; more chains can cross the interface during the bonding process due to the adsorption and entanglement, thus forming a high-strength bond. Nonetheless, this is an overall bonding strength. For the microchannel, more bonding time is required to realize a better sealing performance. On the other hand, more time means an increase in the manufacturing cycle time, which needs to be optimized while ensuring the bonding strength in the following study.

### 3.3. Process Optimization and Leakage Testing

**Figure 9:**
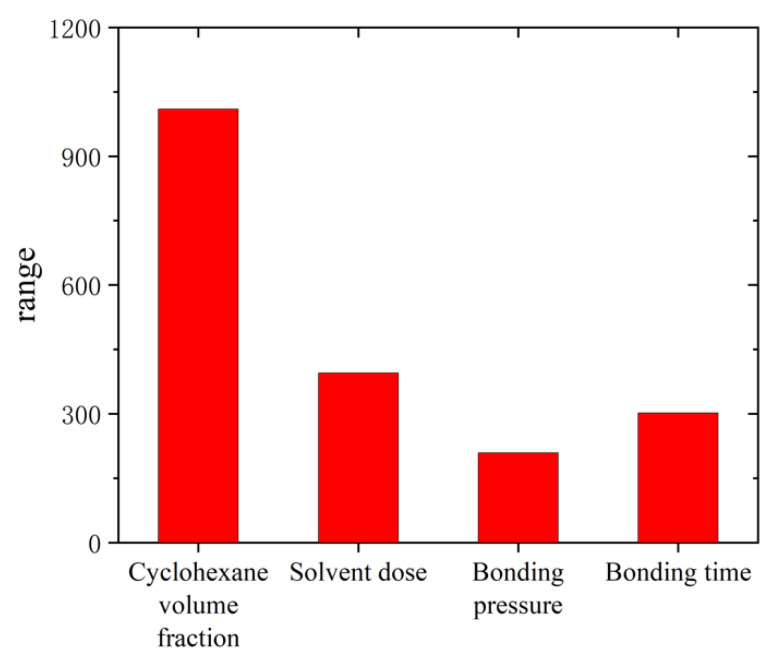
The range change curve under each bonding process.

To clarify the significance of the influence of each factor, the range change curve under each process is presented in Figure 9. It can be seen that the most significant factor influencing each process is the cyclohexane volume fraction. Obviously, the cyclohexane volume fraction is the key factor to affect the bond quality, leading to the variation of bonding strength by affecting the dissolution of the polymer. The solvent dose also affects the dissolution of the polymer by affecting the polymer compared to the cyclohexane volume fraction. Additionally, the bond pressure and time cause less variation to the bonding strength.

**Figure 10:**
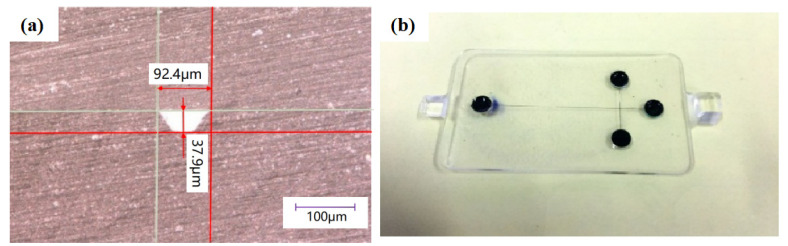
(**a**) Microchannel morphology under the optimized bonding parameters and (**b**) the leakage test.

The optimization analysis of each process was carried out according to the significance of the influence of the process. Therefore, the cyclohexane volume fraction was set to be 70%, with the solvent dose of 1.5 mL, the bonding pressure of 2 MPa, the bonding time of 240 s, and the bonding temperature of 100 °C. Through the above settings, an optimized solvent-assisted in-mold bonding process was set up to seal the microfluidic chip. The bonding strength can be as high as 913 kPa, with the microchannel sizes of 92.4 μm in upper width and 37.9 μm in height, as shown in Figure 10a. When comparing with the designed dimensions of the microchannels, the corresponding deformation in the upper width and height was 7.6% and 5.25%, respectively. With the same bonding parameters in conventional thermal bonding, the corresponding strength was 706 kPa under the bonding temperature of 130 °C. It is demonstrated that the solvent-assistant in-mold bonding strategy can significantly improve the bonding quality. Moreover, the sealing performance was observed by adding an appropriate amount of black ink into the reservoirs. It was found that there was no leakage along the microchannels after the introduction of black ink, as shown in Figure 10b. The surface microchannel sealing performance was excellent and can be used in subsequent studies such as electrophoretic testing.

Compared with conventional thermal bonding methods, the solvent-assisted method can lower the standards for the bonding temperature. Leakage phenomenon occurred by conventional thermal bonding when the bonding temperate was below 120 °C. However, the main disadvantage of the solvent-assisted method would be that the transparency of the treated chip may be affected by the solvent treatment on the COC surface. This can also be revealed from the results of the processed surface in the surface roughness (in Figure 5) and microchannel morphology (in Figure 6). Therefore, the following study will try to eliminate the loss in the optical performance of microfluidic chips that might affect applications such as chemiluminescent detection. In addition, the solvent-induced crystallization behavior should be studied–including the mobile ability of polymer chains at microscale and measurements for the heat distortion and glass transition process for treated polymer–in order to further understand the solvent treatment process and guide parameter selection.

## 4. Conclusions

In this work, a solvent-assisted in-mold bonding method for the COC microfluidic chip is proposed. The organic solvents matched with the COC material were determined by compatibility testing. The influences of the solvent ratio on the chip surface roughness, microchannel morphology, and contact angle were analyzed. With the solvent bonding integrated into the in-mold system, the effects of the solvent volume fraction, solvent dose, bonding pressure, and bonding time on the chip bonding quality and microchannel structure were investigated. The main conclusions are as follows:

(1) The solvent bonding is integrated in the mold, and the bonding of the chip can be realized with the help of the mold temperature during the injection molding process, which shortens the time required to raise the bonding temperature and avoids the problem of coordinating the microchannel morphology and bonding strength at high temperatures.

(2) The solvent cyclohexane needs to be mixed with isopropanol to reduce the dissolution effect. The surface roughness increases with the increase in the cyclohexane volume fraction. Moreover, the surface morphology changes with the increase in cyclohexane volume fraction and the contact angle decreases with the increase in cyclohexane volume fraction. Considering its influence on the surface roughness, contact angle, and surface morphology, the ratio of cyclohexane and isopropanol volume fraction should be set to 7:3.

(3) The solvent-assisted in-mold bonding treatment surface is the cover chip, and the optimized chip bonding process is 70 % cyclohexane volume fraction, 1.5 mL dose, 2 MPa bonding pressure, and 240 s bonding time. At this time, the bonding strength of the chip reaches 913 kPa, the microchannel deformation in upper width and height are 7.6% and 5.25%, respectively, showing a good sealing performance. The solvent-assisted in-mold bonding shows great advantages in terms of bonding temperature and manufacturing cycle time.

## Figures and Tables

**Table 1:** The solubility parameters of selected solvents and COC material.

Materials	Solubility [(J/cm^3^)^1/2^]	Polarity		
		Dispersion Force	Polar Force	Hydrogen Bonding
Cyclohexane	16.7	16.7	0.0	0.2
Isopropanol	23.4	15.5	6.1	16.4
COC	17.7	17.3	3.1	2.1

**Table 2:** The solubility parameters of the mixed solvents.

Serial Number	1	2	3	4	5	6
**Cyclohexane volume fraction/*v/v*%**	100	85	70	55	40	0
**Solubility parameters/(J/cm^3^)^1/2^**	16.7	17.7	18.7	19.7	20.7	23.4

**Table 3:** Single factor experimental design.

Experimental Factors	Level 1	Level 2	Level 3	Level 4	Level 5
**Cyclohexane volume fraction/*v/v*%**	100	85	70	55	40
**Solvent dosage/mL**	0	0.5	1	1.5	2
**Bonding pressure/MPa**	1	2	3	4	5
**Bonding time/s**	120	180	240	300	360

## Data Availability

Not applicable.
